# The relationship among cardiac structure, dietary salt and aldosterone in patients with primary aldosteronism

**DOI:** 10.18632/oncotarget.17505

**Published:** 2017-04-28

**Authors:** Chi-Sheng Hung, Xue-Ming Wu, Ching-Way Chen, Ying-Hsien Chen, Vin-Cent Wu, Che-Wei Liao, Yi-Yao Chang, Ruh-Fang Yen, Ching-Chu Lu, Mao-Yuan M. Su, Kao-Lang Liu, Chin-Chen Chang, Li-Yu Daisy Liu, Kwan-Dun Wu, Yen-Hung Lin, the TAIPAI Study Group

**Affiliations:** ^1^ Department of Internal Medicine, National Taiwan University Hospital and National Taiwan University College of Medicine, Taipei, Taiwan; ^2^ Department of Internal Medicine, Taoyuan General Hospital, Taoyuan, Taiwan; ^3^ Department of Internal Medicine, National Taiwan University Hospital Yun-Lin Branch, Yun-Lin, Taiwan; ^4^ Department of Internal Medicine, National Taiwan University Hospital Hsin-Chu Branch, Hsin-Chu, Taiwan; ^5^ Cardiology Division of Cardiovascular Medical Center, Far Eastern Memorial Hospital, New Taipei City, Taiwan; ^6^ Department of Nuclear Medicine, National Taiwan University Hospital and National Taiwan University College of Medicine, Taipei, Taiwan; ^7^ Department of Medical Imaging, National Taiwan University Hospital and National Taiwan University College of Medicine, Taipei, Taiwan; ^8^ Department of Agronomy, Biometry Division, National Taiwan University, Taipei, Taiwan

**Keywords:** primary aldosteronism, salt intake, left ventricular mass, TAIPAI

## Abstract

Salt intake is highly associated with cardiac structure in patients with primary aldosteronism (PA). We investigated the association among dietary salt intake, aldosterone and left ventricular mass in patients with PA. We enrolled 158 patients with PA and 158 patients with essential hypertension. We measured 24-hour urinary sodium (UNa) and aldosterone (UAldo) level and echocardiography parameters. In patients with PA, the UAldo level was positively correlated with left ventricular mass index (LVMI; r=0.231, p=0.007). The UNa level was not linearly correlated with left ventricular structural parameters in patients with PA. To test if UNa has a non-linear relationship with LVMI among patients with PA, we categorized the participants according to the tertile of UNa (low, median, and high tertile). PA patients with medium tertile of UNa had significant lower LVMI than the other two groups (LVMI: 144.1 ± 42.9, 121.1 ± 33.4, and 136.7 ± 32.8 g/m2, from the lowest to the highest tertile of Una; analysis of variance p=0.006, post-hoc p <0.05). Multifactor analysis of variance confirmed this finding after adjustment for clinical parameters. Post-hoc analyses revealed that the high UNa tertile was associated with higher left ventricular end-diastolic volume compared with medium UNa tertile; while the low UNa tertile was associated with higher mean wall thickness compared with medium UNa tertile. The findings imply the reasons for increased LVMI may be different in patients with the highest and lowest UNa tertile. In conclusion, the medium tertile of 24-hour UNa is associated with lowest LVMI in patients with PA.

## INTRODUCTION

Primary aldosteronism (PA), characterized by excessive production of aldosterone, is now considered to be one of the main causes of secondary hypertension [1]. The reported prevalence of PA in patients with hypertension ranges from 5-15% [2]. Patients with PA have high rates of cardiovascular complications, including left ventricular hypertrophy (LVH), heart failure, myocardial infarction, stroke and atrial fibrillation [3, 4].

LVH, or increased LV mass, is more common in patients with PA than in those with essential hypertension (EH) or other types of secondary hypertension [5]. LVH develops early in the course of PA, and is preceded by other end-organ damage [6]. LVH is also an independent risk factor for cardiovascular mortality [7]. Patients with PA have a higher rate of LVH and an inappropriately high LV mass for the degree of workload and blood pressure compared to patients with EH [8]. Aldosterone has been reported to be a major cause of inappropriate increases in LV mass in patients with PA, and has been shown to directly cause cardiac structural damage, including cardiomyocyte hypertrophy and myocardial fibrosis, independently of the effect of blood pressure or angiotensin II [9, 10]. Rossi et al reported 323 patients with PA, in whom the LV mass improved to the level of optimally treated EH after adrenalectomy or treatment with a mineralocorticoid receptor antagonist during long-term follow-up [11].

In patients with EH, dietary sodium intake and plasma aldosterone concentration have been reported to be independently associated with LV mass [12, 13]. In patient with PA, however, the relative contributions of sodium and aldosterone to LVH are less clear. A recent study on patients with PA suggested a positive correlation between dietary sodium and LV mass [14], although the patients with PA in their study had relatively high salt intake compared to the average daily salt intake worldwide [15], and it is unclear whether the same results would have been found in PA patients with normal or low sodium intake. Moreover, a large clinical observation study reported a U-shaped relationship between sodium intake and mortality [16]. A recent animal study also reported that lower salt intake could still induce LV hypertrophy and fibrosis in the presence of excess aldosterone [17]. Hence, it is possible that the relationship between sodium intake and LV mass is non-linear.

We hypothesized that in patients with PA, the relationship between salt intake and LV mass would be weakest in those with a medium sodium intake, and that the relationship between aldosterone and LV mass would be positive and linear. We conducted this cross-sectional study to evaluate the relationships between 24-hour urinary aldosterone and sodium excretion and LV structural parameters using echocardiography.

## MATERIALS AND METHODS

### Patients

This cross-sectional study enrolled 158 patients diagnosed with PA from October 2006 to March 2010, all of whom were registered in the Taiwan Primary Aldosteronism Investigation (TAIPAI) database. This database was constructed for quality assurance in one medical center (National Taiwan University Hospital, Taipei, Taiwan), and its three affiliated hospitals around Taiwan (National Taiwan University Hospital Yun-Lin branch, Yun-Lin, southern Taiwan; Far-Eastern Memorial Hospital, Taipei; and Taoyuan General Hospital, Taoyuan, central Taiwan) [18]. Another 158 patients with EH were enrolled for comparison. The medical history of the subjects, including demographic data and current medications, was recorded. The diagnosis of EH was made by exclusion according to standard algorithms based on clinical history, and biochemical investigations of all detectable forms of secondary hypertension. All of the patients were evaluated with echocardiography and 24-hour urine collection for sodium and aldosterone concentrations. The 24-hour urine collection was performed under the subjects’ usual diet reflecting salt appetite. This study complied with the Declaration of Helsinki and was approved by the Institutional Review Board of National Taiwan University Hospital (Taipei, Taiwan). Informed consent was obtained from all participants before inclusion in the study.

### Laboratory measurements

A 24-hour urine sample with the first urine of the day included was collected from all subjects and refrigerated until analysis. The 24-hour urinary sodium and aldosterone amount (UNa and UAldo, respectively) were calculated by multiplying the urine sodium or aldosterone concentration by the daily urine amount. When interpreting the results of 24-hour urine collection, we assessed the adequacy of collection by quantifying the 24-hour urine creatinine excretion value, defined as a value between 15 and 20 mg/kg of body weight. The urine samples were stored in plastic containers at 4°C.

The plasma aldosterone concentration (PAC) was measured using radioimmunoassays with commercial kits (Aldosterone Maia Kit, Adaltis Italia S.P.A., Bologna, Italy) [19]. Plasma renin activity (PRA) was calculated as the generation of angiotensin-I *in vitro* using a commercially available radioimmunoassay kit (Cisbio, Bedford, MA). All antihypertensive medications were discontinued for at least 21 days before measuring plasma PRA and PAC. Diltiazem and/or doxazosin was administered to control markedly high blood pressure when required.

#### Diagnostic criteria for PA

Fulfillment of the following three conditions was taken to confirm a diagnosis of aldosteronism: (1) autonomous excess aldosterone production evidenced with an aldosterone to renin ratio (ARR) > 35; (2) a TAIPAI score > 60% [20]; (3) post-saline loading PAC > 10 ng/dl, or PAC/PRA > 35 (ng/dL)/(ng/mL/h) shown in a post captopril/losartan test, or PAC > 6 ng/dL as indicated by a fludrocortisone suppression test [21].

### PA subtype identification

Aldosterone-producing adenoma was identified on the basis of the following four conditions: (1) autonomous excess aldosterone production evidenced with an ARR > 35, a TAIPAI score > 60% [20], and post-saline loading PAC > 10 ng/dl; (2) adenoma evidenced on a computed tomography (CT) scan for pre-operative evaluation; (3) lateralization of aldosterone secretion at adrenal vein sampling or during dexamethasone suppression NP-59 SPECT/CT [22]; (4) pathologically proven adenoma after adrenalectomy for the patients who underwent surgery, and the subsequent emergence of either a cure pattern of hypertension without anti-hypertensive agents or improvement in hypertension, potassium, PAC, and PRA [21, 23, 24].

Idiopathic hyperaldosteronism was defined on the basis of the following four criteria: (1) autonomous ex-cess aldosterone production evidenced with an ARR > 35, a TAIPAI score > 60% [20], and post-saline loading PAC > 10 ng/dl; (2) evidence of bilateral diffuse enlargement as seen on a CT scan for pre-operative evaluation; (3) non-lateralization of aldosterone secretion at AVS or during dexamethasone suppression NP-59 SPECT/CT [22]; (4) evidence of diffuse cell hyperplasia reported in following pathology studies for those who underwent surgery.

#### Echocardiography

All echocardiography was performed using a Hewlett-Packard 5500 ultrasound system with an S3 transducer (1.0-3.0 MHz). Transthoracic echocardiographic images were obtained in fundamental imaging modes. Two-dimensional, M-mode, Doppler and tissue Doppler ultrasonography were performed in each patient, and the dimensions of the chamber, wall thickness and left ventricular ejection fraction (M-mode) were measured according to the guidelines of the American Society of Echocardiography [25].

LVMI was derived from echocardiography according to the formula reported by Devereux and Reichek: [ LV mass = 1.04 x [(septal thickness + LV end-diastolic diameter + posterior wall thickness)^3^ - (LV end-diastolic diameter)^3^]-13.6] [26]. Predicted LVMI was estimated using a previously derived equation: predicted LVM = 55.37+ 6.64 x height ^2.7^+ 0.64 x stroke work-18.07 x gender (where gender was scored as male=1 and female=2) [27]. Left ventricle volume was calculated using Tericholz's formula, and stroke work was calculated as systolic blood pressure (in mmHg) x stroke volume x 0.0144 [8]. Inappropriate LVMI or excessive LV mass compared to the predicted LVMI, was defined as: measured LVMI – predicted LVMI. LVH was defined according to Devereux's criteria: LVMI ≥ 134 g/m^2^ in men and 110 g/m^2^ in women [28].

### Statistical analysis

All continuous variables were expressed as mean ± standard deviation if normally distributed. Non-normally distributed variables were expressed as median and interquartile range. Comparison of continuous variables between the two groups were preformed using the Student's t-test (normally distributed) or Wilcoxon rank sum test (non-normally distributed). Differences between proportions were assessed using the chi-square test. Urinary aldosterone concentration was log-transformed before the correlation study due to non-normality, which was determined by the Kolmogorov-Smirnov test.

Pearson's correlation tests were first used to analyze the correlations between LVMI and 24-hour UAldo or UNa level. Partial correlations were then used to adjust for age, gender and systolic blood pressure. To test the non-linear relationship between urinary aldosterone or sodium and LVMI, we categorized the patients into tertiles according to the UAldo or UNa level (low, medium, and high tertiles). Multifactor ANOVA was performed to test the effect of UAldo or UNa tertile on LVMI, with adjustments for age, sex, mean blood pressure and duration of hypertension. An interaction term of UAldo and UNa was included to test the interaction. According to the LVMI formula, LVMI was determined by both LV wall thickness and end-diastolic volume. Therefore, if the association between UAldo or UNa and LVMI was significant, we further analyzed the association between UAldo or UNa and wall thickness and end-diastolic volume.

ANOVA was used separately for the patients with PA and those with EH. Tukey's honest significant difference test was used for post-hoc analysis. All statistical analyses were performed using Stata/SE software version 11.2 for Windows (StataCorp LP, Texas) and R 2.14.0 software (R Foundation for Statistical Computing, Vienna, Austria).

## RESULTS

One hundred and fifty-eight PA patients and 158 EH patients were enrolled in this study. The mean age of all participants was 51.8 ± 12.4 years, and the baseline clinical characteristics are shown in Table 1. The mean UNa level was 154.1 ± 82.1 mmol/day in all participants (154.4 ± 91.8 mmol/day in the PA group and 153.8 ± 70.6 mmol/day in the EH group). The median 24-hour UAldo level was 13.76 and 8.7 μg per day in the PA and EH groups, respectively. Patients with PA had a higher PAC, 24-hour UAldo level, longer duration of hypertension, higher systolic blood pressure, more medications for hypertension and lower serum potassium level and PRA. The serum sodium concentration and 24-hour UNa level were similar between the two groups. The patients with PA had a thicker LV wall thickness, higher LVMI, and higher inappropriate LVMI compared to the patients with EH (Table 2).

**Table 1:** Baseline characteristics and echocardiographic findings of study participants

	Primary aldosteronism (n=158)	Essential hypertension (n=158)	P value
Age, year, mean(SD)	51.8(11.6)	51.8(13.1)	0.978
Sex, male, n(%)	68(43%)	87(55.1%)	0.033
Body mass index, kg/m^2^, mean(SD)	25.1(3.5)	26.8(12.1)	0.096
Duration of hypertension, year, mean(SD)	8.5(7.8)	6.2(7.1)	0.007
Systolic blood pressure, mmHg, mean(SD)	151.8(20.6)	145.1(21.6)	0.005
Diastolic blood pressure, mmHg, mean(SD)	89.3(12.5)	86.7(13.0)	0.075
APA, n(%)	124(79.0%)	-	-
**Medications**			
ARB or ACEI, n(%)	24(15.1%)	20(12.7%)	0.472
Calcium channel blocker	104(67.1%)	91(58.0%)	0.115
Thiazide, n(%)	62(39.2%)	17(10.8%)	<0.001
Beta-blocker, n(%)	73(47.1%)	36(22.8%)	<0.001
Alpha-blocker, n(%)	33(21.3%)	23(14.6%)	0.120
Direct vasodilator, n(%)	4(2.6%)	7(4.4%)	0.374
**Biochemistry data**			
Serum sodium, mEq/L, mean(SD)	139.9(8.2)	139.8(2.2)	0.892
Serum potassium, mEq/L, mean(SD)	3.5(0.8)	4.2(0.4)	<0.001
Plasma aldosterone concentration*, ng/dL, median (25^th^, 75^th^ percentile)	41.4(28.6, 63.2)	35.3(24.6, 49.9)	<0.001
Plasma renin activity*, ng/ml.hr, median (25^th^, 75^th^ percentile)	0.27(0.07, 0.64)	2.36(1, 7.48)	<0.001
Aldosterone to renin ratio*, median (25^th^, 75^th^ percentile)	141.6(50.3, 683.5)	12.9(6.4, 45.2)	<0.001
Urinary aldosterone* μg/24 hours, median (25^th^, 75^th^ percentile)	13.76(7.1, 27.5)	8.7(5.5, 15.3)	<0.001
Urinary sodium, mmol/24 hours, mean(SD)	154.4(91.8)	153.8(70.6)	0.946
Glomerular filtration rate (ml/min/1.73 m^2^, by MDRD equation), mean(SD)	85.4(25.9)	84.7(25.6)	0.809

ACEI: angiotensin converting enzyme inhibitor; ARB: angiotensin II Receptor Blocker; MDRD: Modification of Diet in Renal Disease

*Expressed as mean and interquartile range

**Table 2:** Echocardiographic parameters of study participants

	Primary aldosteronism (n=158)	Essential hypertension (n=158)	P value
Mean wall thickness, mm, mean(SD)	11.2(1.8)	10.8(1.6)	0.033
LVEDD, mm, mean(SD)	47(4.6)	46.5(4.9)	0.406
LVESD, mm, mean(SD)	28.6(3.9)	28.1(4.5)	0.264
LVEDV, mL, mean(SD)	103.9(24.0)	101.1(26.2)	0.325
LVESV, mL, mean(SD)	32.1(11.3)	30.9(12.1)	0.403
LVEF, %, mean(SD)	69.1(6.4)	70.3(6.5)	0.103
LVMI, g/m^2^, mean(SD)	133.9(37.7)	122.3(36.9)	0.007
Inappropriate LVMI, g/m^2^, mean(SD)	45.6(30.4)	36.7(26.6)	0.007
LVH, n(%)	94(59.5%)	64 (40.5%)	0.001

LVEF: left ventricular ejection fraction; LVEDD: left ventricular end-diastolic diameter; LVEDV: left ventricular end-diastolic volume; LVESD: left ventricular end-systolic diameter; LVESV: left ventricular end-systolic volume; LVH: left ventricular hypertrophy: LVMI, left ventricular mass index.

The correlations between 24-hour UAldo level and PAC were moderate in the patients overall, and in the PA and EH groups (Pearson's correlation coefficient: 0.304, p<0.001; 0.215, p = 0.011; and 0.377, p < 0.001, respectively). There was no significant correlation between 24-hour UNa level and PAC in the patients overall or in the PA and EH groups.

In the patients with PA, the correlations between 24-hour UAldo level and LV mass were significant (LVMI r=0.231, p=0.007; inappropriate LVMI, r=0.188, p=0.029) (Table 3). These correlations remained significant after adjusting for age, sex and systolic blood pressure. The correlations between 24-hour UNa level and LV structural parameters were not significant in the patients with PA. In the patients with EH, the 24-hour UAldo level was not correlated with LV structural parameters, while the 24-hour UNa level was correlated with inappropriate LVMI (r=0.185, p=0.027). However, the correlations were not statistically significant after adjusting for age, sex and systolic blood pressure (Table 3).

**Table 3:** The correlations between 24-hour urinary sodium or aldosterone and LV structural parameters among (1) patients with primary aldosteronism or (2) patients with essential hypertension

		Primary aldosteronism				Essential hypertension			
LV		Crude		Adjusted*		Crude		Adjusted*	
Structural parameters		Correlation coefficient	p-value	Correlation coefficient	p-value	Correlation coefficient	p-value	Correlation coefficient	p-value
LVMI	UNa	−0.024	0.769	−0.087	0.292	0.117	0.161	0.132	0.122
	UAldo	0.231	0.007	0.216	0.013	0.074	0.368	0.078	0.351
Inappropriate LVMI	UNa	−0.035	0.668	−0.097	0.239	0.185	0.027	0.141	0.097
	UAldo	0.188	0.029	0.178	0.041	0.092	0.274	0.119	0.160

LVMI: left ventricular mass index; UAldo: 24-hour urinary aldosterone amount; UNa: 24-hour urinary sodium amount

*Adjusted by age, sex, systolic blood pressure

**Figure 1:**
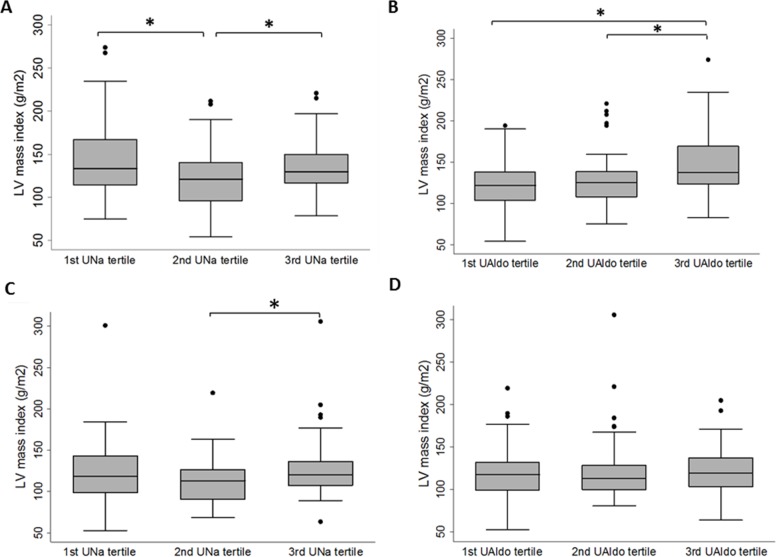
Box plots of left ventricular (LV) mass index by 24-hour urinary sodium (UNa) tertiles or 24-hour urinary aldosterone (Ualdo) tertiles among patients with primary aldosteronism **(A, B)** or essential hypertension **(C, D)**. The thick horizontal line represents the median LV mass index, the box represents interquartile range, whiskers represent 95% confidence intervals, and dots represent outlying observations. *p < 0.05 in post-hoc analysis.

To test if there were non-linear relationships between UNa or UAldo and LVMI, we plotted LVMI according to UNa or UAldo tertile (Figure 1A and 1B, for the patients with PA; Figure 1C and 1D, for the patient with EH). The LVMI values of the patients with low, medium, and high UNa tertiles were 144.1 ± 42.9, 121.1 ± 33.5, and 136.7 ± 32.8 g/m^2^, respectively, for the PA group (p=0.006, see also Supplementary Table 2), and 121.2 ± 41.6, 113.3 ± 27.2 and 129.6 ± 38.6 g/m^2^, respectively, for the EH group (p=0.102). The LVMI values of the patients with low, medium, and high UAldo tertiles were 122.1 ± 31.2, 129.1 ± 34.4, and 146.8 ± 40.2 g/m^2^, respectively, for the PA group (p=0.003) (see also Supplementary Table 4), and 119.1 ± 32.7, 122.9 ± 38.6, and 121.0 ± 30.6 g/m^2^, respectively, for the EH group (p=0.866). These results indicated that among patients with PA, the medium UNa tertile group had a lower LVMI compared to the high and low tertile groups. The clinical characters of PA patients with low, medium, and high UNa tertiles were shown in Supplementary Table 1.

Among the patients with PA, multifactor ANOVA revealed significantly different LVMI values in the UNa and UAldo tertiles after adjusting for covariates (Table 4). There were no interactions between UNa and UAldo tertiles. Post-hoc analysis with Tukey's honest significant difference test revealed significant differences between the medium and high UNa tertiles and the medium and low tertiles, but not between the low and high tertiles (Supplementary Table 4).

**Table 4:** Multi-factor ANOVA for LVMI by UAldo and UNa tertiles among patients with primary aldosteronism

	Primary aldosteronism N = 158	
Source	F statistic	p-value
Age	0.28	0.598
Sex	13.15	<0.001
Hypertension duration	1.98	0.162
Mean blood pressure	5.78	0.018
UAldo, tertile	6.11	0.003
UNa, tertile	7.29	0.001
UAldo*UNa	0.25	0.907

UAldo: 24-hour urinary aldosterone amount; UNa: 24-hour urinary sodium amount

**Figure 2:**
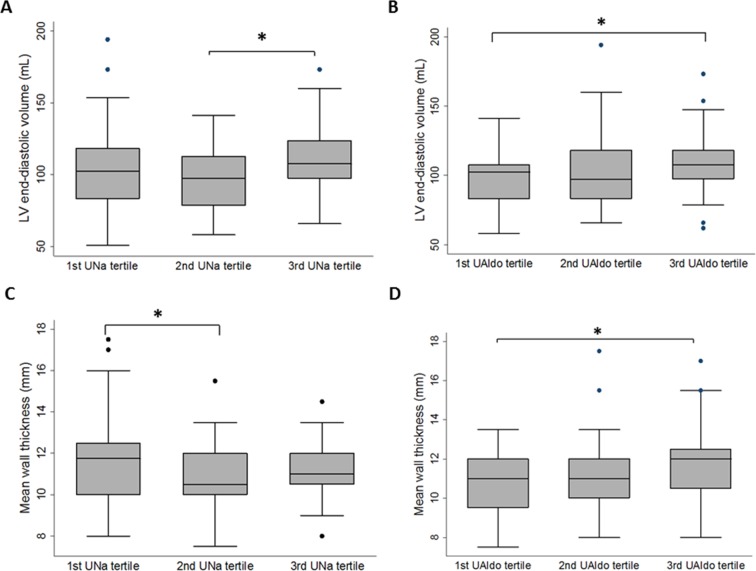
Box plots of left ventricular (LV) end-diastolic volume or mean wall thickness by 24-hour urinary sodium (UNa) tertiles or 24-hour urinary aldosterone (Ualdo) tertiles among patients with primary aldosteronism **(A)** LV end-diastolic volume, by 24-hour UNa tertile. **(B)** LV end-diastolic volume, by 24-hour UAldo tertile. **(C)** Mean wall thickness, by 24-hour UNa tertile. **(D)** Mean wall thickness, by 24-hour UAldo tertile. The thick horizontal line represents the mean value, the box represents interquartile range, whiskers represent 95% confidence intervals, and dots represent outlying observations. *p < 0.05 in post-hoc analysis.

Because LV mass depends on the LV mean wall thickness (MWT) and LV end-diastolic volume (LVEDV), we tested the relationship between these two LV structural parameters and 24-hour UNa or UAldo tertile among the patients with PA. The results showed that MWT and LVEDV were significantly different among the three UNa tertiles after adjusting for age, sex, hypertension duration, blood pressure and tertile of UAldo (Supplementary Table 3). Post-hoc analysis revealed that the LVEDV was significantly higher in the high UNa tertile compared to the medium UNa tertile, while the MWT was significantly higher in the low UNa tertile compared to the medium UNa tertile (Figure 2A and 2C; Supplementary Table 4). In contrast, the MWT and LVEDV were only borderline different among the three UAldo tertiles after adjusting for age, sex, hypertension duration, blood pressure and tertile of UNa (Figure 2B and 2D; Supplementary Table 4).

## DISCUSSION

Our results demonstrated that in patients with PA, the 24-hour UAldo level was positively associated with LVMI, while the medium UNa tertile was associated with a lower LVMI compared to the low or high UNa tertiles. These differences in LVMI can partly be explained by the LVEDV in the medium versus high UNa tertiles and by the MWT in the low versus medium UNa tertiles.

The relationships between dietary sodium intake, aldosterone and LV dimension have been previously investigated. Pimenta et al recruited 21 patients with PA and 21 patients with EH matched by age, gender and blood pressure, and found that urinary sodium was positively correlated with LV mass in the patients with PA [14]. The difference between their results and ours is probably due to the difference in sodium intake. The urinary sodium level in their PA patients was 216.1 ± 65.6 mmol/d, compared to 154.4 ± 91.8 mmol/d in our PA patients. The urinary sodium level in our study is closer to the world mean dietary sodium level (158 mmol/d) [29] and a prior report in Taiwan (122 mmol/d) [30]. Therefore, their data depicted the relationship between the sodium level among PA patients with higher sodium intake (corresponding to the medium and high tertiles in our data) and LV structure. One recent study investigated the association between dietary salt intake and cardiac change after treatment in patients with PA [31]. The mean urinary sodium level of patients with PA in that study (149 mmol/d) is closer to ours. However, the mean urinary sodium of low tertile in our study (75.8 mmol/d) is much lower than theirs (100 mmol/d). Furthermore, the major LV mass difference in that study is between medium (LV mass index 49.0 ± 9.6 g/m^2^) and higher tertile (LV mass index 55.9 ±14.3 g/m^2^). There is only minimal difference of LV mass between patients with medium and lower tertile (LV mass index 46.4 ±7.3 g/m^2^). Our results expand the current knowledge about the relationship between the sodium level in PA patients and a wider range of sodium intake and LV structure.

The finding that a medium tertile of sodium intake was associated with a lower LV mass has not been reported before in patients with PA and EH. However, U- or J-shaped relationships between sodium intake and hypertension or cardiovascular events have been reported. In a prior study in Taiwan, 24-hour urinary sodium level was curvilinearly associated with incident hypertension among 1520 middle-aged and elderly participants during 7.9 year of follow-up [30]. The participants with a urinary sodium level between 84-122 mmol/day had the lowest rate of incident hypertension compared to the participants with lower or higher sodium levels. In the Prospective Urban Rural Epidemiology (PURE) study, which included 101,945 participants, a J-shaped association between sodium intake and cardiovascular disease or death was demonstrated [16]. A recent pooled analysis from four large prospective studies also supported this finding [32]. Although these studies were carefully conducted, a reverse causation cannot be fully excluded. A prior pre-clinical study reported that low dietary sodium intake can still induce LV hypertrophy and fibrosis in the presence of excess aldosterone. Hattori et al used uninephrectomized rats with low or high salt intake and aldosterone mini-pump implantation to investigate cardiac damage [17]. The results showed that in the setting of low salt intake and excess aldosterone, substantial cardiac remodeling and diastolic dysfunction occurred. In addition, blood pressure was not elevated in the low salt intake group, although the concentration of reactive oxygen species was significantly increased. These findings imply that excess levels of aldosterone can induce hypertrophy and fibrosis in low-salt conditions via inflammation without the contribution of hypertension. Nevertheless, low salt intake has been associated with elevated plasma catecholamine and aldosterone concentrations among patients with EH [33, 34]. Further studies are warranted to investigate whether an elevated level of catecholamine contributes to a higher LV mass in patients with PA with lower salt intake.

Our study revealed that the increased LVMI in high UNa tertile was associated with increased LVEDV and increased LVMI in low UNa tertile was associated with increased MWT in PA patients. The detail mechanisms whether salt/aldosterone combination promotes cardiac dimension and structure change are still unclear. Both aldosterone and high salt intake induce fluid retention and expand intravascular volume [14]. In the study by Pimeta et al, PA patients have much higher LVEDV than EH patients (115.1 ± 29.3 ml for PA patients vs. 80.6 ± 22.2 ml for EH patients, p<0.001) [14]. However, PA patients also have much higher UNa value than EH patients (216.1 ± 65.6 mmol/d for PA patients vs.155.5 ± 65.6 mmol/d for EH patients, p = 0.004). In that study, high salt intake in hyperaldosterone status leads to the great difference of LVEDV between PA and EH patients. In the presented study, PA patients with high Una tertile had higher LVEDV than PA patients with medium UNa tertile, which also demonstrate the effect of high salt intake on LVEDV in hyperaldosterone status. In the other aspect, increased LVMI in low UNa tertile was associated with increased MWT in PA patients in the presented study. Reduced salt intake is associated with increased plasma norepinephrine concentrations, and the effects are more stronger in hypertensive patients [33, 34]. In addition, elevated plasma norepinephrine concentrations are associated with increased LV wall thickness and concentric LV hypertrophy [35]. Therefore, increased plasma norepinephrine concentrations may be one of the reasons to explain the increased MWT in PA patients with low UNa tertile.

The relationship between sodium intake and LVMI may also be modified by genetic factors. In a previous clinical study evaluating the relationship between angiotensin-converting enzyme (ACE) genotypes and LVMI, a medium sodium intake was associated with a lower LVMI compared to a higher or lower sodium intake in Italian offspring with ACE DD and DI genotypes but not in the II genotype or in Slavic offspring [36]. In the study by Isaji et al, there is a significant correlation between urinary sodium and LVMI in patients with CYP11B2 C/C, but not in CYP11B2 C/T and CYP11B2 T/T [37]. However, the incidence of CYP11B2C/C is only 7.5 % in Taiwanese [38]. This might also weaken the relation between UNa and LVMI in the presented study.

There are several limitations to this study. First, we use a single 24-hour urine collection to estimate dietary salt as previous studies [14, 39]. However, regarding the day-to-day variability of salt intake, the average derived from multiple measurement of 24-hour urinary sodium excretion are more accurate compared with a single 24-hour measurement. The variability in the urinary sodium measurement may underestimate the association between sodium intake and cardiac dimensions. Second, some anti-hypertensive medication may have influence on left ventricular structure and urinary sodium excretion. Further studies are need to clarify their effects. Third, because of the cross-sectional design, a clear causation could not be established based on our results. Therefore, recommendations on dietary salt intake in patient with PA cannot be given based on our results. Fourth, compared with other population-based studies in patients with EH, our sample size was relatively small, and this may have prevented us from showing a relationship between LVMI and urinary sodium or aldosterone. Further large-scale studies are needed to confirm our results. Last, the duration of hypertension was different between PA and EH groups. Further studies with same hypertension duration in both groups are needed in the future.

## CONCLUSION

In the patients with PA, a medium 24-hour UNa level was associated with a lower LVMI compared to a higher or lower UNa level, and the 24-hour UAldo level was positively associated with LVMI.

## SUPPLEMENTARY TABLES







## Appendix

Membership of the Taiwan Primary Aldosteronism Investigation (TAIPAI) Study Group: Che-Hsiung Wu, MD (Chi-Taz hospital, PI of Committee); Vin-Cent Wu, MD, PhD (NTUH, PI of Committee); Yen-Hung Lin, MD, PhD (NTUH, PI of Committee); Yi-Luwn Ho, MD, PhD (NTUH, PI of Committee); Hung-Wei Chang, MD, PhD (Far Eastern Hospital, PI of Committee); Lian-Yu Lin MD, PhD (NTUH, PI of Committee); Fu-Chang Hu, MS, ScD, (Harvard Statistics, Site Investigator); Kao-Lang Liu, MD (NTUH, PI of Committee); Shuo-Meng Wang, MD (NTUH, PI of Committee); Kuo-How Huang, MD, PhD (NTUH, PI of Committee); Yung-Ming Chen, MD (NTUH, PI of Committee); Chin-Chi Kuo; MD (Yun-Lin, PI of Committee), Chin-Chen Chang, MD (NTUH, PI of Committee); Shih-Cheng Liao, MD (NTUH, PI of Committee); Ruoh-Fang Yen, MD, PhD (NTUH, PI of Committee); and Kwan-Dun Wu, MD, PhD (NTUH, Director of Coordinating Center).
