# Supplementary material for: The relationship among cardiac structure, dietary salt and aldosterone in patients with primary aldosteronism
Source: Oncotarget. 2017 Apr 28;8(42):73187–97. doi: 10.18632/oncotarget.17505 (PMC5641205; doi:10.18632/oncotarget.17505)
Supplement: Supplementary file 1 [file oncotarget-08-73187-s001.pdf]

The relationship among cardiac structure, dietary salt and aldosterone in patients with primary aldosteronism

SUPPLEMENTARY TABLES

Supplementary Table 1: Baseline characteristics in patients with primary aldosteronism by 24-hour urinary sodium tertile.

See Supplementary File 1

Supplementary Table 2: LV structural parameters in patients with primary aldosteronism by 24-hour urinary sodium tertile

	1st tertile	2nd tertile	3rd tertile	p
**N**	**53**	**52**	**53**	
Mean wall thickness, mm, mean(SD)	11.6(2.0)	10.9(1.8)	11.3(1.4)	0.189
LVEDD, mm, mean(SD)	47.2(5.3)	45.4(4.1)	48.4(4)	0.004
LVESD, mm, mean(SD)	28.5(4.9)	28(3.1)	29.3(3.5)	0.273
LVEDV, mL, mean(SD)	105.2(27.8)	95.7(19.9)	110.8(21.4)	0.004
LVESV, mL, mean(SD)	31.8(14.4)	30.8(8.4)	33.3(9.8)	0.546
LVEF, %, mean(SD)	69.9(7.1)	68.2(5.5)	69.3(6.5)	0.382
LVMI, g/m^2, mean(SD)	144.1(42.9)	121.1(33.5)	136.7(32.8)	0.006
Inappropriate LVMI, g/m^2, mean(SD)	54.3(37.3)	39.1(26.3)	43.7(24.9)	0.034
LVH, n(%)	40(75.5%)	24(46.2%)	30(56.6%)	0.008

LVEF, left ventricular ejection fraction; LVEDD, left ventricular end-diastolic diameter; LVEDV, left ventricular end-diastolic volume; LVESD, left ventricular end-systolic diameter; LVESV, left ventricular end-ayatolic volume; LVH, left ventricular hypertrophy; LVMI, left ventricular mass index

Supplementary Table 3: Multi-factor ANOVA for left ventricular end-diastolic volume or left ventricular mean wall thickness by UAldo and UNa tertiles among patients with primary aldosteronism

	Primary aldosteronism, N = 158			
	Left ventricular end-diastolic volume		Left ventricular mean wall thickness	
**Source**	**F statistic**	**p-value**	**F statistic**	**p-value**
Age	0.86	0.356	0.89	0.347
Sex	22.34	<0.001	29.68	<0.001
Hypertension duration	3.2	0.076	0.71	0.403
Mean blood pressure	4.62	0.034	5.26	0.024
UAldo, tertile	2.57	0.08	2.66	0.074
UNa, tertile	6.57	0.002	4.03	0.02
UAldo*UNa	0.94	0.441	1.16	0.334

UAldo, 24-hour urinary aldosterone amount; UNa, 24-hour urinary sodium amount

Supplementary Table 4: LVMI, MWT and LVEDV by tertile of Una or Ualdo among patients with primary aldosteronism.

See Supplementary File 2
