# Supplementary material for: The relationship among cardiac structure, dietary salt and aldosterone in patients with primary aldosteronism
Source: Oncotarget. 2017 Apr 28;8(42):73187–97. doi: 10.18632/oncotarget.17505 (PMC5641205; doi:10.18632/oncotarget.17505)
Supplement: Supplementary file 2 [file oncotarget-08-73187-s002.docx]

**Supplementary Table 1. Baseline characteristics in patients with primary aldosteronism by 24-hour urinary sodium tertile**

|  | **1^st^ tertile** | **2^nd^ tertile** | **3^rd^ tertile** | **p** |
| --- | --- | --- | --- | --- |
| **N** | **53** | **52** | **53** |  |
| Age, year, mean(SD) | 52.5(12.2) | 49.7(12.1) | 53.2(10.3) | 0.271 |
| Sex, male, n(%) | 14(26.4%) | 22(42.3%) | 32(60.3%) | 0.001 |
| Body mass index, kg/m^2^, mean(SD) | 24.6(3.7) | 25.1(3.5) | 25.7(3.2) | 0.244 |
| Duration of hypertension, year, mean(SD) | 8.3(6.8) | 6.2(6.8) | 11.1(8.9) | 0.004 |
| Systolic blood pressure, mmHg, mean(SD) | 153.9(22.6) | 148.1(19.8) | 153.4(19) | 0.286 |
| Diastolic blood pressure, mmHg, mean(SD) | 90.3(11.5) | 89.6(12.1) | 87.9(14) | 0.594 |
| APA, n(%) | 42(79.3%) | 40(76.9%) | 40(75.5%) | 0.924 |
| **Medications** |  |  |  |  |
| ARB or ACEI, n(%) | 9(17.0%) | 4(8%) | 11(21.2%) | 0.173 |
| Calcium channel blocker | 39(73.6%) | 27(54%) | 38(73.1%) | 0.057 |
| Thiazide, n(%) | 21(39.6%) | 18(36%) | 23(44.2%) | 0.696 |
| Beta-blocker, n(%) | 24(45.3%) | 24(48.0%) | 25(48.1%) | 0.948 |
| Alpha-blocker, n(%) | 16(30.2%) | 7(14%) | 10(19.2%) | 0.121 |
| Direct vasodilator, n(%) | 1(1.9%) | 0(0%) | 3(5.8%) | 0.171 |
| **Biochemistry data** |  |  |  |  |
| Serum sodium, mEq/L, mean(SD) | 141.1(3.5) | 137.9(13.3) | 140.8(3) | 0.107 |
| Serum potassium, mEq/L, mean(SD) | 3.3(0.6) | 3.5(0.7) | 3.7(1) | 0.075 |
| Plasma aldosterone concentration*, ng/dL, median (25^th^, 75^th^ percentile) | 45.4(31.3, 76.23) | 40.1(26.8, 69,8) | 38.7(29, 49.8) | 0.122 |
| Plasma renin activity*, ng/ml.hr, median (25^th^, 75^th^ percentile) | 0.26(0.11, 0.59) | 0.42(0.06, 0.73) | 0.24(0.07, 0.54) | 0.544 |
| Aldosterone to renin ratio*, median (25^th^, 75^th^ percentile) | 148.4(78.5, 732.3) | 109.5(38.3, 870.3) | 138.3(64.2, 555.2) | 0.615 |
| Urinary aldosterone* μg/24 hours, mean(SD) | 26.5(21.5) | 22.8(18.3) | 19.3(12.6) | 0.167 |
| Urinary sodium, mmol/24 hours, mean(SD) | 75.8(28.4) | 137.8(15.6) | 246.3(95.7) | <0.001 |
| Glomerular filtration rate (ml/min/1.73 m^2^, by MDRD equation), mean(SD) | 87.2(28.2) | 87.9(24.2) | 81.1(25) | 0.328 |

APA: aldosterone-producing adenoma; ACEI: angiotensin converting enzyme inhibitor; ARB: angiotensin II Receptor Blocker; MDRD: Modification of Diet in Renal Disease.

*Expressed as mean and interquartile range
