# Supplementary material for: The relationship among cardiac structure, dietary salt and aldosterone in patients with primary aldosteronism
Source: Oncotarget. 2017 Apr 28;8(42):73187–97. doi: 10.18632/oncotarget.17505 (PMC5641205; doi:10.18632/oncotarget.17505)
Supplement: Supplementary file 3 [file oncotarget-08-73187-s003.docx]

**Supplementary Table 4: LVMI, MWT and LVEDV by tertile of Una or Ualdo among patients with primary aldosteronism**

|  | **Primary aldosteronism, N = 158** | | | **p-values, unadjusted / adjusted*** | | | |
| --- | --- | --- | --- | --- | --- | --- | --- |
|  |  |  |  | **ANOVA** | **Post-hoc analysis** | | |
|  | **1^st^ tertile** | **2^nd^ tertile** | **3^rd^ tertile** |  | **1^st^ vs 2^nd^** | **1^st^ vs 3^rd^** | **2^nd^ vs 3^rd^** |
| LVMI, g/m2 |  |  |  |  |  |  |  |
| By UNa tertile | 144.1(42.9) | 121.1(33.5) | 136.7(32.8) | 0.006 / 0.001 | 0.005 / 0.001 | 0.568 / 0.487 | 0.078 / 0.041 |
| By UAldo tertile | 122.1(31.2) | 129.1(34.4) | 146.8(40.2) | 0.003 / 0.003 | 0.056 / 0.557 | 0.001 / 0.001 | 0.028 / 0.028 |
| MWT, mm |  |  |  |  |  |  |  |
| By UNa tertile | 11.6(2.0) | 10.9(1.8) | 11.3(1.4) | 0.189 / 0.020 | 0.161 / 0.031 | 0.655 / 0.123 | 0.594 / 0.853 |
| By UAldo tertile | 10.8(1.5) | 11.2(1.8) | 11.7(1.8) | 0.051 /0.074 | 0.540 / 0.466 | 0.039 / 0.016 | 0.340 / 0.251 |
| LVEDV, mL |  |  |  |  |  |  |  |
| By UNa tertile | 105.2(27.8) | 95.7(19.9) | 110.8(21.4) | 0.004 / 0.002 | 0.099 / 0.051 | 0.435 / 0.341 | 0.003 / 0.001 |
| By UAldo tertile | 97.5(19.0) | 102.2(26.6) | 109.0(22.5) | 0.060 / 0.080 | 0.582 / 0.506 | 0.047 / 0.022 | 0.344 / 0.262 |

*p-value, adjusted: multifactor ANOVA with age, sex, hypertension duration, mean blood pressure, tertile of urinary aldosterone, tertile of urinary sodium and interaction term of urinary aldosterone and urinary sodium in model

LVEDV: left ventricular end-diastolic volume; LVMI: Left ventricular mass index; MWT: left ventricular mean wall thickness; UAldo: 24-hour urinary aldosterone amount; UNa: 24-hour urinary sodium amount
